# A Real-World Study of Patient Characteristics and Clinical Outcomes in *EGFR* Mutated Lung Cancer Treated with First-Line Osimertinib: Expanding the FLAURA Trial Results into Routine Clinical Practice

**DOI:** 10.3390/cancers16061079

**Published:** 2024-03-07

**Authors:** Hollis Viray, Andrew J. Piper-Vallillo, Page Widick, Emmeline Academia, Meghan Shea, Deepa Rangachari, Paul A. VanderLaan, Susumu S. Kobayashi, Daniel B. Costa

**Affiliations:** 1 Division of Medical Oncology, Department of Medicine, Beth Israel Deaconess Medical Center, Boston, MA 02215, USA; hviray@bidmc.harvard.edu (H.V.); andrew.piper@lahey.org (A.J.P.-V.); drangach@bidmc.harvard.edu (D.R.); skobayas@bidmc.harvard.edu (S.S.K.); 2 Division of Hematology/Oncology, Department of Medicine, Lahey Hospital & Medical Center, Burlington, MA 01805, USA; 3 Department of Pathology, Beth Israel Deaconess Medical Center, Boston, MA 02215, USA; pvanderl@bidmc.harvard.edu

**Keywords:** lung cancer, EGFR, osimertinib, real-world, time to treatment discontinuation, survival

## Abstract

**Simple Summary:**

Cancer drug approvals are based on clinical trials with strict inclusion and exclusion criteria, and more often than not, the patient population encountered in real-world settings is different (with additional comorbidities or different patient-disease characteristics) than the one that led to regulatory approval. Oral inhibitors of the EGFR oncogene are approved for use in *EGFR* mutated lung cancers. We sought to evaluate if the EGFR inhibitor osimertinib would perform in real-word populations in a manner expected by the registration trial of this anti-cancer agent. We were able to identify cases in our real-world cohort that had durations of both cancer control and overall survival that were in line with the data from the registration clinical trial named FLAURA. The real-world data presented here support the translation of results from clinical trials to routine clinical care for targeted therapy in *EGFR* mutated lung cancer but also highlight the need for clinical trials that are more inclusive.

**Abstract:**

Osimertinib is a tyrosine kinase inhibitor of the epidermal growth factor receptor (EGFR) that is used for first-line therapy in *EGFR* mutated non-small cell lung cancer (NSCLC) based on the results of the randomized FLAURA trial (ClinicalTrials.gov number NCT02296125). We performed a retrospective analysis of baseline characteristics and clinical outcomes in 56 real-world patients treated with osimertinib. In total, 45% of patients were determined to be FLAURA-eligible and 55% were FLAURA-ineligible based on the published inclusion/exclusion criteria of the aforementioned trial. For clinical outcomes, the median osimertinib time to treatment discontinuation (TTD) for all patients was 16.9 months (95% CI: 12.6–35.1), whereas the median TTD was 31.1 months (95% CI: 14.9–not reached) in the FLAURA-eligible cohort and the median TTD was 12.2 months (95% CI: 8.1–34.6 months) in the FLAURA-ineligible cohort. Re-biopsy at acquired resistance disclosed both on- and off-target mechanisms. The most common therapies following osimertinib included local therapies followed by post-progression osimertinib, platinum-doublet chemotherapy with or without osimertinib, and osimertinib combinatory targeted therapies. The median overall survival for all patients was 32.0 months (95% CI: 15.7–not reached), the median survival was not reached for the FLAURA-eligible cohort, and it was 16.5 months for the FLAURA-ineligible cohort. Our data support the use of osimertinib in real-word settings and highlight the need for designing registration trials that are more inclusive of patient/disease characteristics seen in routine clinical practice. It is yet to be determined if the use of evolving first-line EGFR inhibitor combination strategies (either platinum-doublet chemotherapy plus osimertinib or amivantamab plus lazertinib) will similarly translate from clinical trials to real-word settings.

## 1. Introduction

Activating driver mutations of the epidermal growth factor receptor (*EGFR*) gene are present in approximately 15–30% of patients diagnosed with non-small cell lung cancer (NSCLC) [1,2,3]. Oral targeted therapy with EGFR tyrosine kinase inhibitors (TKIs) became the first-line treatment for patients with metastatic and unresectable NSCLC with activating *EGFR* mutations since the late 2000s [4,5,6]. Osimertinib is a third-generation, mutation-specific, covalent EGFR TKI that is active against common EGFR mutants (exon 19 deletions/indels and exon 21 L858R), less common EGFR mutants (exon 18 G719X, exon 20 S768I, exon 20 A763_Y764insFQEA, and exon 21 L861Q, plus others), and the first/second-generation resistant EGFR-T790M mutated protein [6,7]. Osimertinib cemented its status as the preferred first-line systemic therapeutic option for *EGFR* mutated (*EGFR*m) NSCLC following the publication, in 2018, of the FLAURA trial (ClinicalTrials.gov number NCT02296125) comparing osimertinib to the first-generation EGFR TKIs gefitinib or erlotinib in patients whose tumors harbored *EGFR*-exon 19 deletions/indels or *EGFR*-L858R [8]. Owing to its improved mutation selectivity, decreased side-effect profile, and superior intracranial activity, osimertinib was shown in the FLAURA clinical trial to lead to both improved progression-free survival (PFS) and overall survival (OS) compared to the first-line use of gefitinib or erlotinib [8,9]. This seminal trial established the median PFS of 18.9 months and the median OS of 38.6 months attributed to osimertinib in the first-line setting [8,9]. Multiple combinatory approaches of third-generation EGFR TKIs with additional therapies—such as osimertinib plus platinum-based chemotherapy (FLAURA2 trial, NCT04035486) [10] or lazertinib plus amivantamab (MARIPOSA trial, NCT04487080) [11]—have been recently reported and may lead to newer approaches for the first-line management of *EGFR*m NSCLC.

Prior studies have evaluated real-world experiences with Osimertinib, but the majority focus on the experience of patients treated in the second-line, third-line, and beyond settings with tumors harboring *EGFR*-T790M [12,13,14,15]. Many of these real-world studies only focus on patterns of progression and mechanisms of resistance following initial progression on osimertinib [16,17,18,19]. There is a paucity of data that evaluate the real-world translation of the FLAURA clinical trial results into routine clinical practice, especially as related to both correlates of PFS plus OS in cohorts of patients that match or do not match the inclusion criteria of the aforementioned trial.

Herein, we describe a retrospective analysis of a real-world experience treating patients with advanced *EGFR*m NSCLC receiving first-line osimertinib. This study seeks to compare patient and tumor characteristics with those included in the FLAURA trial as well as the real-world treatment response, real-world mechanisms of resistance, subsequent therapeutic selection, and survival metrics.

## 2. Materials and Methods

### 2.1. Study Design

**Figure 1:**
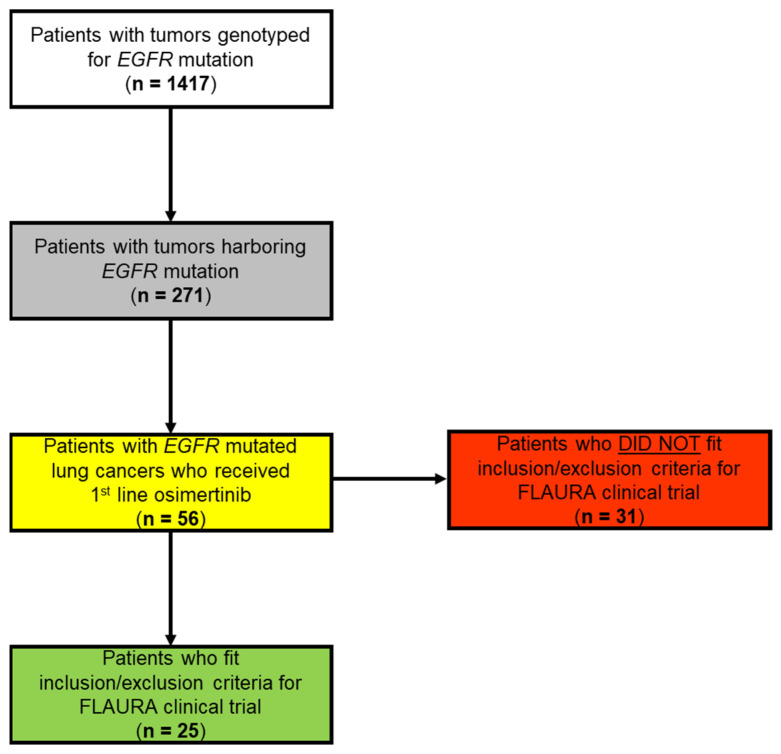
Diagram of Beth Israel Deaconess Medical Center (BIDMC) Real-world Cohort with Allocation to FLAURA Clinical Trial Eligibility. Identification of cases to be included in a detailed retrospective medical chart review based on the tumor EGFR mutation status and the use of first-line osimertinib, with designations of FLAURA clinical trial eligibility within the real-world BIDMC cohort. Patients were determined to be FLAURA-ineligible if they either did not meet all FLAURA inclusion criteria or they met a protocol-specified FLAURA exclusion criterion (as per references [8,9]).

We conducted a retrospective cohort study on patients with unresectable or metastatic NSCLC with *EGFR* mutations identified at the time of diagnosis who were treated with first-line osimertinib therapy. The patients were treated at a National Cancer Institute designated cancer center and academic medical center, Beth Israel Deaconess Medical Center (BIDMC) in Boston, Massachusetts, United States of America. Medical records from patients were included in the study if the patient had an NSCLC with an *EGFR* mutation identified until the cut-off of February 2021 and if the patient had initiated osimertinib as the first-line therapy, as detailed in the Figure 1. This study was conducted in accordance with a research protocol approved by the Institutional Review Board (IRB) of BIDMC, as described in prior studies from our group [20,21,22,23,24]. The informed consent of individual participants was waived per the IRB approval of the use of de-identified medical record data.

### 2.2. Cohort Selection and Procedures

A retrospective review of the medical record was completed for patients included in the study. Patient demographic information, baseline clinical and histopathological characteristics, and next-generation sequencing data were collected. Additionally, the treatment duration and the response of osimertinib and OS were collected along with the mechanism of resistance and subsequent therapeutic treatment initiation. Based on the patient characteristics and tumor profile, patients were determined to be either FLAURA-eligible or FLAURA-ineligible by using the published FLAURA trial inclusion and exclusion criteria in the publicly available protocol [8,9]. Time to treatment discontinuation (TTD) is a pragmatic end point that is defined as the date of starting a medication to the date of treatment discontinuation or death and has been proposed as a real-world evidence correlate of PFS [25,26]. The TTD of osimertinib in this study was defined as the months between osimertinib initiation and discontinuation or the need to add an additional therapy beyond initial osimertinib or death. The radiographic response was not obtained prospectively but retrospectively. The surrogate response parameter of the disease control rate (DCR) to osimertinib was extrapolated based on documentation by the treating provider in the available medical record and radiology reports of the absence of radiographic/clinical progression. OS was calculated from the initiation of osimertinib to death. Data were censored if the outcome (either TTD or OS) had not been reached at the time of analyses or if follow-up data were not available.

### 2.3. Statistical Analysis

The statistical analysis was performed using Stata (StataCorp LLC, College Station, TX, USA). Descriptive statistics were generated for the overall population as well as the FLAURA “eligible” and “ineligible” cohorts. Median TTD and OS were calculated using Kaplan–Meier analyses for the overall cohort as well as for cohorts stratified by FLAURA eligibility. The 95% confidence interval (95% CI) values for each median were obtained. Hazard ratios (HRs) for TTD and OS were calculated using the unadjusted Cox proportional hazards model comparing the FLAURA-eligible versus FLAURA-ineligible cohorts, with *p* < 0.05 defined as the threshold for rejecting the null hypothesis. Multivariate regression analysis was performed to adjust for several baseline clinicopathologic characteristics including age, smoking status, performance status, and self-reported Asian ancestry.

## 3. Results

### 3.1. Baseline Patient Characteristics

In total, 56 patients were included in this cohort study (Figure 1). The median age was 68 years (range 35–102 years) and the cohort was 61% female (Table 1). The baseline characteristics were compared to those of the FLAURA study intervention arm (eligible cases that were treated with osimertinib), which had a total of 279 patients [8,9]. Our real-world cohort had a higher percentage of self-reported patients of the White race (62%) and Black race (9%) compared to the FLAURA cohort, which included 64% patients with self-reported Asian ethnicity [8,9]. Notably, 25% of our real-world patients had Eastern Cooperative Oncology Group (ECOG) performance statuses of two or higher, while patients with World Health Organization (WHO) performance statuses of only zero or one were eligible for the FLAURA trial [8,9]. BIDMC’s real-world cohort contained similar amounts of metastatic versus locally advanced NSCLC patients as FLAURA, with a similar distribution of patients with visceral and/or central nervous system (CNS) metastatic disease (Table 1). While the FLAURA study only included patients with the most common *EGFR* activating mutations (exon 19 deletions/indels or L858R mutation), BIDMC’s real-world cohort also included eight (15%) patients with less common *EGFR* mutations in their tumors, including *EGFR*-G719X, *EGFR*-L861Q, and *EGFR*-A763_Y764insFQEA (Table 1).

### 3.2. FLAURA Eligibility Evaluation

Following the collection of baseline patient and tumor histopathologic features plus comprehensive genomic profiling data, patients in this real-world cohort were determined to be either FLAURA-eligible or FLAURA-ineligible based on whether or not they met all the trial inclusion/exclusion criteria based on the FLAURA trial protocol [8,9]. Of the 56 total patients included in the study, 25 (45%) were determined to be FLAURA-eligible and 31 (55%) were determined to be FLAURA-ineligible based on at least one exclusion criterion (Figure 1 and Table 2). Within the FLAURA-ineligible patients, the most common exclusion criteria included cardiac risk factors, an inadequate performance status, less common *EGFR* mutations, and renal dysfunction, among others (Table 2).

### 3.3. Treatment Response and Survival

Regarding the real-world treatment response and survival, we again compared our clinically assessed simple response to osimertinib to published response rates from the FLAURA trial [8]. Our real-world assessment of DCR was similar to those published in the FLAURA trial. We report a DCR of 81% in BIDMC’s real-world cohort (Table 3). Only three patients (5%) had primary progression, as defined by available records, while on first-line osimertinib (Table 3).

**Figure 2:**
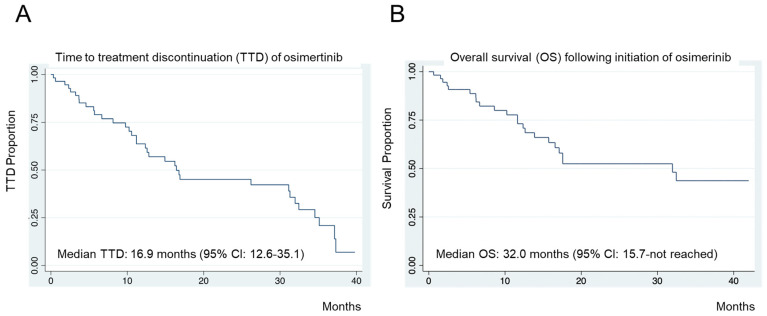
Real-World Cohort Clinical Outcomes. (**A**) Time to treatment discontinuation (TTD) of osimertinib-treated cases in the BIDMC real-world cohort. (**B**) Overall survival (OS) from the start of osimertinib in BIDMC’s real-world cohort.

For each patient in the real-world cohort, we calculated their time to osimertinib discontinuation (using TTD) as the number of months between osimertinib initiation and discontinuation or the need to add additional therapy beyond the initial osimertinib or death. In the overall BIDMC real-world cohort, the median TTD was 16.9 months (95% CI: 12.6–35.1 months) (Figure 2A). We also calculated individual-level survival times from the initiation of osimertinib to death. The median OS in BIDMC’s real-world cohort was 32.0 months (95% CI: 15.7–not reached [NR] months) (Figure 2B). Both the real-world median TTD and median OS were within the 95% CI of the reported outcomes in the FLAURA trial that reported a median PFS of 18.9 months (95% CI: 15.2–21.4 months) and a median OS of 38.6 months (95% CI: 34.5–41.8 months) [8,9].

**Figure 3:**
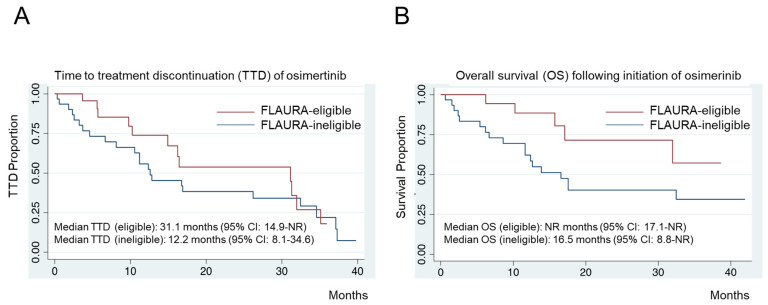
Real-World Cohort Clinical Outcomes Stratified by FLAURA Trial Eligibility. (**A**) Time to treatment discontinuation (TTD) of osimertinib-treated cases in the BIDMC real-world cohort. (**B**) Overall survival (OS) from the start of osimertinib in BIDMC’s real-world cohort. TTD and OS were stratified by FLAURA-eligible and FLAURA-ineligible status allocation. NR, not reached.

**Figure 4:**
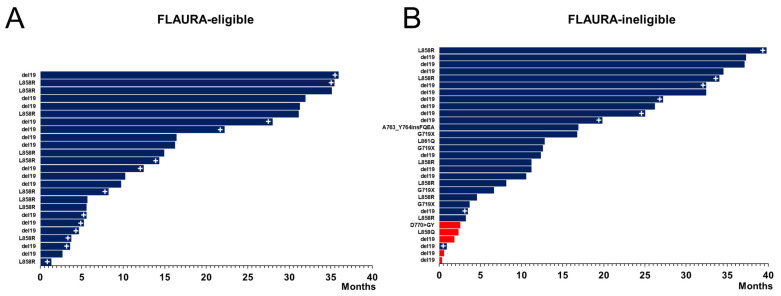
Real-World Cohort Swimmers’ Plots of Osimertib Time to Treatment Discontinuation. (**A**) Individual case time to treatment discontinuation (TTD) of osimertinib-treated cases in the BIDMC real-world cohort for the FLAURA-eligible cohort. (**B**) Individual case TTD of osimertinib-treated cases in the BIDMC real-world cohort for the FLAURA-ineligible cohort. The type of *EGFR* mutation is identified for each case, with “del19” representing *EGFR*-exon 19 deletions or EGFR-exon 19 indels. The dark blue-filled bars indicate cases that had disease control from osimertinib, while the red-filled bars indicate cases that had primary disease progression on osimertinib, early death while receiving osimertinib, or a lack of assessment of disease control within the initial 6 weeks of osimeritnib prior to discontinuation (additional details in Table 3). The white + symbol indicates cases that continued on the initial osimertinib at the time of data cut-off (i.e., ongoing first-line osimertinib therapy).

Within our real-world cohort, TTD and OS were also stratified into FLAURA-eligible versus FLAURA-ineligible patients (Figure 3). The median TTD and OS were improved in the FLAURA-eligible subgroup versus those in the FLAURA-ineligible subgroup. The median TTD in the FLAURA-eligible group was 31.1 months (95% CI: 14.9–NR months), while the median TTD in the FLAURA-ineligible group was 12.2 months (95% CI: 8.1–34.6 months). The individual-level TTD is provided in the Swimmer’s plot (Figure 4). Similarly, the median OS in the FLAURA-eligible group was NR (95% CI: 17.1–NR months), while the median OS in the FLAURA-ineligible group was 16.5 months (95% CI: 8.8–NR months). 

Utilizing an unadjusted Cox proportional hazards model, we observed a shorter OS for the FLAURA-ineligible group when compared to the FLAURA-eligible group (HR 0.36; 95% CI: 0.13–0.96, *p* = 0.04), but there was no significant difference between the FLAURA eligibility-stratified groups when adjusted for age, smoking status, performance status, CNS disease at baseline, and self-reported Asian ancestry using multivariate regression analysis. The same unadjusted and adjusted Cox proportional hazards model was used to evaluate TTD by FLAURA eligibility-stratified groups, but no statistical significance was noted. None of the aforementioned individual adjustment characteristics in the overall cohort were significantly associated with differences in clinical outcomes. 

### 3.4. Mechanisms of Resistance

At the time of the data analysis cut-off for clinically defined progression, 30 patients in BIDMC’s real-world cohort had retrospective data supporting a classification of clinical/radiographic progressive disease by the treating provider on first-line osimertinib (Table 4). Cases were reported as having the progression of the visceral disease burden only, only CNS progression, or both (Table 4).

Repeat comprehensive genomic profiling at the time of progression was performed for 20 patients in our real-world cohort to assess the mechanisms of resistance to osimertinib. Some patient-derived biopsies (liquid or tissue) had identified actionable mechanisms of resistance, including off-target *MET* amplification and on-target *EGFR*-C797S mutation (Table 4). 

### 3.5. Post-Progression Therapy Selection 

We also collected data on patients’ systemic therapy choice following initial disease progression on osimertinib. Of the 18 patients for whom we had data on their post-progression treatment, 14 (78%) continued osimertinib post-progression plus or minus additional systemic therapies (Table 4). Five patients continued single-agent osimertinib following localized therapy for oligo-progressive disease, and an additional four patients with actionable mechanisms of resistance continued osimertinib with the addition of a supplementary targeted therapy agent. The most common systemic therapy offered was platinum-doublet chemotherapy (Table 4). Seven patients started platinum-doublet chemotherapy (mostly carboplatin and pemetrexed) following osimertinib progression, including five patients who continued osimertinib with platinum-doublet chemotherapy use (Table 4). 

## 4. Discussion

We describe real-world experience with first-line osimertinib use in metastatic *EGFR*m NSCLC at an academic medical center to expand on the existing literature of real-world studies of osimertinib and similar targeted therapies. Our retrospective review revealed that over half (55%) of the patients included in our real-world setting would not have been eligible for inclusion in the original FLAURA registration trial [8,9]. Our cohort included a quarter of patients with ECOG performance statuses of two or greater and additional cases with increased comorbidities, most notably those with pre-existing cardiac toxicity and/or those at an increased risk of cardiac toxicity (Table 2). 

Our real-world cohort had a median TTD for osimertinib of 16.9 months (95% CI: 12.6–35.1 months) and a median OS of 32.0 months (95% CI: 15.7–NR months), which are comparable to the median PFS median of 18.9 months (95% CI: 15.2–21.4 months) and the median OS of 38.6 (95% CI: 34.5–41.8 months) reported in the FLAURA trial [8,9]. This finding is supported by the previously reported real-world studies of osimertinib. For example, the OSI-FACT study reporting outcomes in patient with *EGFR*m NSCLC treated with first-line osimertinib conveyed a median PFS of 20.5 months (95% CI: 18.6–NR months) [17], and the FLOWER study reporting first-line osimertinib outcomes in Italy reported a median PFS of 18.9 months (95% CI: 11.2–26.7 months) and a median TTD of 25.3 months [16]. An additional Swiss cohort study reported a median time to osimertinib failure of 22.9 months (95% CI: 17.6–29.3 months) [18]. Interestingly, no real-world studies of first-line osimertinib, including our BIDMC cohort, reported significantly worse outcomes regarding treatment duration and effectiveness when compared to the seminal clinical trial-obtained values of the FLAURA trial [8,9]. 

Taken together, these results are reassuring regarding the effectiveness of osimertinib in a more general and less selective patient population and stand in agreement with the translation of clinical trials to real-word studies of other targeted therapies for NSCLC [27,28,29,30,31,32,33] but in contrast to real-world studies involving other treatment modalities, such as chemotherapy and immunotherapy. In our own BIDMC cohort of advanced cases of NSCLC treated with immune checkpoint inhibitors, we observed clinical outcomes—in part dictated by baseline performance status—that were significantly inferior to those reported in the seminal approval trials of the same agents [23]. Similar reports on the use of single-agent immune checkpoint inhibitors have been reported by other groups [34,35], and the same type of efficacy–effectiveness lag into a real-word setting has been shown for chemo-immunotherapy [36,37]. 

We were pleasantly surprised to find that, particularly in our FLAURA-eligible subgroup of patients (Figure 3), the median TTD was numerically longer than the median PFS reported in the FLAURA trial [8]. This suggests that fit patients with the most common *EGFR* mutated tumors fair as well or better in real-world settings than clinical trial candidates and/or that clinicians in the real world may be opting to continue osimertinib treatment past the traditional parameters of radiographic progression that often define PFS [25].

We also sought to better characterize the mechanisms of resistance to osimertinib and the choice of post-progression systemic therapy in this real-world setting. A Swiss cohort study of post-osimertinib progression reported that approximately 60% of osimertinib progression is categorized as oligo-progressive disease [18]. For patients requiring second-line systemic therapy, the majority of patients in that study received platinum-doublet chemotherapy without the continuation of osimertinib [18]. This is consistent with published guidelines and recommendations from both the National Comprehensive Cancer Network and the European Society for Medical Oncology [38,39]. Regarding the use of second-line therapeutic selection in our cohort, most patients continued osimertinib even after initial progression. In general, patients with oligo-progressive disease are often treated with localized therapies and then continue on osimertinib for systemic control [16,17,18]. Our study also highlights the potential for continuing osimertinib plus additional systemic therapy, such as platinum-doublet chemotherapy (Table 4), and this is a strategy that beckons further evaluation with randomized clinical trials. 

Multiple prior studies have published frequencies of mechanisms of resistance in *EGFR*m NSCLC treated with osimertinib [40,41,42]. Notably, most tumors with progression on osimertinib have no identified genomic mechanism of resistance using routine clinical assays, and even fewer have an actionable mechanism of resistance that allows for additional targeted therapy [42]. Most often, these include off-target *MET* amplification or on-target *EGFR*-C797S mutation [43,44]. We report similar findings to prior published studies regarding the mechanism of resistance to Osimertinib, with the majority of patients in BIDMC’s real-world cohort lacking an identifiable and actionable mutation (Table 4). The mechanisms of resistance to osimertinib and other EGFR TKIs are heterogeneous and often not identified by the current genomic technologies used in clinical practice [45]. Preclinical and translational studies have shown that drug-tolerant persistent cells that remain viable following EGFR TKIs, through multiple mechanisms including the activation of the AXL receptor tyrosine kinase, provide a nidus for eventual clinical/radiographic osimertinib acquired resistance [40,46]. Ongoing clinical studies are attempting to overcome osimertinib resistance based on aforementioned preclinical studies. It is also important to highlight that the activity of osimertinib in the treatment-naïve setting is quite heterogeneous in both preclinical models and clinical settings in *EGFR*m tumors harboring less common EGFR mutations. We and others have described in detail the clinical efficacy of osimertinib against *EGFR*-G719X, *EGFR*-L861Q, *EGFR*-A763_Y764insFQEA, *EGFR*-exon 19 insertions, and *EGFR*-S768I mutated tumors and the lack of the clinical activity of osimertinib in most *EGFR*-exon 20 insertion mutated tumors [1,3,4,5,6,20,24].

The limitations of this study include the fact that it is a retrospective and single-institution study without a centralized review of tumor responses for calculating PFS, and we used TTD as a surrogate real-work parameter to accommodate for this limit [25]. In addition, our cohort size may have limited identifying one specific parameter (be it performance status, smoking, or age, among others) that correlated with survival outcomes. It was expected—and confirmed in our analyses—that FLAURA trial eligibility encompassed multiple prognostic parameters that eventually dictate OS, and this likely explains the group (herein defined as FLAURA-eligible) that most benefited from osimertinib in our real-world setting (Figure 3). Another limitation is that trends in treatment selection, particularly after progression, are reflective of our institutional practice, the availability of clinical trials, and culture. However, this study still adds valuable insight into real-world treatment durations, outcomes, and post-progression trends after first-line osimertinib in *EGFR*m NSCLC. 

As the clinical management of patients with metastatic *EGFR*m NSCLC evolves in the future, this study provides important information regarding real-world experience with Osimertinib, particularly as both first- and second-line systemic therapies change in 2024 and beyond. It is possible that recent clinical trials in the first-line setting have the potential to replace osimertinib monotherapy as the preferred first-line regimen in select patients with *EGFR*m NSCLC [10,47,48]. For example, the MARIPOSA clinical trial of the first-line EGFR-MET antibody amivantamab plus the third-generation EGFR TKI lazertinib in *EGFR*m NSCLC reported an improved median PFS of 23.7 months compared to that of osimertinib, with OS data favoring amivantamab plus lazertinib as well [47,48]. Similarly, the FLAURA2 trial also reported an improved PFS when treating with first-line platinum-pemetrexed chemotherapy plus osimertinib versus that of osimertinib monotherapy [10], although the OS results are too immature to determine if this chemotherapy plus osimertinib strategy should be incorporated widely or narrowly for select cases [10].

These trials, among others, are likely to lead to new regulatory approvals worldwide and possibly change treatment guidelines for front-line therapy in *EGFR*m NSCLC. Thus, it is increasingly important to understand the risk/benefit of each possible regimen in the real-world setting to best tailor therapy to each patient.

## 5. Conclusions

We report a single academic center’s experience with real-world osimertinib use in *EGFR*m NSCLC, of which more than half of the patients would have been ineligible for inclusion in the practice-changing FLAURA trial [8,9]. The median TTD and 95% CIs in the overall cohort were comparable to the median PFS reported in the FLAURA study [8]. The outcomes in our real-world FLAURA-eligible patients were numerically higher than those of the FLAURA study participants. 

Our data support the use of osimertinib in real-word settings and highlight the need for registration trials that are more inclusive of patient and disease characteristics seen in routine clinical practice. 

It is yet to be determined if the use of evolving first-line EGFR inhibitor combination strategies (either chemotherapy plus osimertinib or amivantamab plus lazertinib [10,11,47,48]) will similarly translate from clinical trial to real-word *EGFR*m NSCLC settings.

## Figures and Tables

**Table 1:** Baseline Patient Characteristics of the Real-World BIDMC Cohort versus those of the FLAURA Trial.

Characteristic	FLAURA Osimertinib Cohort (n = 279)	BIDMC Osimertinib Cohort (n = 56)
Age—years		
Median	64	68
Range	26–85	35–102
Male sex—no. (%)	101 (36)	22 (39)
Female sex—no. (%)	178 (64)	34 (61)
Race/Ethnicity—no. (%)		
White (non-Asian)	101 (36)	35 (62)
Black (non-Asian)	0 (0)	5 (9)
Asian	174 (62.5)	16 (29)
Other	4 (1.5)	0 (0)
Smoking status—no. (%)		
Never (0 pack-years)	182 (65)	34 (61)
Current	8 (3)	3 (5)
Former ^	89 (32)	19 (34)
Performance Status *		
0	112 (40)	14 (25)
1	167 (60)	28 (50)
2	0 (0)	8 (14)
3	0 (0)	5 (9)
4	0 (0)	1 (2)
Histologic type—no. (%)		
Adenocarcinoma	275 (98.5)	54 (96)
Squamous	0 (0)	1 (2)
Mixed histology	4 (1.5)	1 (2)
Overall disease classification—no. (%)		
Metastatic	264 (94.5)	54 (96)
Locally advanced	14 (5)	2 (4)
Missing data	1 (0.5)	0 (0)
Metastases—no. (%)		
CNS metastases	53 (19)	12 (21)
*EGFR* mutation type—no. (%)		
Exon 19 deletions/indels	175 (63)	32 (57)
L858R	104 (37)	16 (28)
G719X ^^	0 (0)	4 (7)
L861Q	0 (0)	1 (2)
Exon 20 A763_Y764insFQEA	0 (0)	1 (2)
Exon 20 D770>GY	0 (0)	1 (2)
L858Q	0 (0)	1 (2)

The FLAURA trial details available in references [8,9]; * FLAURA trial used WHO performance status, while our real-world (RW) cohort used ECOG performance status; ^ Includes two patient who snorted tobacco; ^^ Includes one co-mutation of *EGFR*-G719A+E709K; no., number.

**Table 2:** Real-world Patients Meeting Exclusion Criteria based on the FLAURA Trial.

Exclusion Criteria per FLAURA Trial Protocol #	BIDMC Cohort, No.
**Squamous cell carcinoma histology**	1
***EGFR* mutation type different than *EGFR*-exon 19 deletion/indel or *EGFR*-L858R**	8
**WHO/ECOG performance status not 0 or 1**	14
**Major surgery within 4 weeks of osimertinib**	2
**Spinal cord compression**	0
Symptomatic or unstable brain metastases *	5
**Comorbid conditions**	
Uncontrolled hypertension	6
Active bleeding diatheses	2
Refractory nausea/vomiting, chronic gastrointestinal illness, inability to swallow, or previous significant bowel resection	1
**Cardiac criteria**	
Resting corrected QTc > 470 ms	5
Any clinically important ECG abnormality ^	8
Factors that increase the risk of arrhythmic events ^^	16
**History of lung fibrosis or radiation pneumonitis requiring steroids**	0
**Concurrent second malignancy**	0
**Inadequate hematologic reserve or organ function**	
Absolute neutrophil count < 1.5 K/µL	0
Platelet count < 100 K/µL	0
Hemoglobin < 90 g/L	1
Alanine aminotransferase > 2.5× upper limit of normal	0
Aspartate aminotransferase > 2.5× upper limit of normal	0
Total bilirubin > 1.5× upper limit of normal if no liver metastases or >3× upper limit of normal with a history of Gilbert’s Syndrome (indirect) or liver metastases	1
Creatinine > 1.5× upper limit of normal concurrent with creatinine clearance < 50 mL/min	5

# FLAURA trial details available in references [8,9]; * Symptomatic central nervous system (CNS) disease burden; ^ Arrhythmia, conduction abnormality, or morphologic abnormality such as complete left bundle branch block, third-degree heart block, second-degree heart block, or PR interval > 250 ms; ^^ History of heart failure, hypokalemia, congenital long QT syndrome, family history of long QT syndrome or first-degree relative with sudden death under age 40, or QTc prolonging medications; no., number.

**Table 3:** BIDMC Real-World Treatment Response Pattern.

Simple Response—No. (%)	FLAURA Cohort (n = 279)	BIDMC Cohort (n = 56)
Disease control rate (DCR)	270 (97%)	51 (91%)
Primary Progression	3 (1%)	3 (5%)
Death	0 (0%)	2 (4%) *
Unable to evaluate	6 (2%)	0 (0%)

* Both deaths determined to be unrelated to osimertinib or lung cancer but occurring prior to the 6-week assessment time point. DCR calculated as complete response, partial response, and stable disease for ≥6 weeks within FLAURA clinical trial references [13,15], while it was collated as a lack of physician-determined clinical plus radiographic progression in the real-world BIDMC cohort.

**Table 4:** Mechanisms of Resistance and Second-Line Therapy Following Progression on Osimertinib.

	Number of Cases
Progression on first-line osimertinib at data cut-off (n = 56)	
Present	30
Absent	24
Unknown	2
Pattern of Progression (n = 30)	
CNS only progression	9
Visceral only progression	16
Both	2
Unknown	3
Resequencing Modality (n = 30)	
Liquid Biopsy	10
Tissue Biopsy	12
Not Done/Unknown	8
Mechanism of Resistance (n = 20)	
*EGFR*-C797S	1
*MET* Amplification	3
Small Cell Lung Cancer Transformation	0
Pre-Existing/Non-Actionable Mutations	12
None Detected/Test Inconclusive	3
Unknown	1
Post-Osimertinib Progression Therapy (n = 18)	
Platinum doublet chemotherapy	1
Platinum doublet chemotherapy + Osimertinib	5
Platinum doublet chemotherapy + Immunotherapy	1
Osimertinib Post-Progression Following Local Therapy	5
Osimertinib + MET-Targeted TKI	4
First-generation EGFR TKI (Erlotinib or Gefitinib)	2

CNS, central nervous system; TKI, tyrosine kinase inhibitor.

## Data Availability

The data presented in this study are available on request from the corresponding author but limited to de-identified information approved by the Beth Israel Deaconess Medical Center Committee on Clinical Investigations.
